# Vessels supplying blood to the encephalon of the alpaca (*Vicugna pacos*)

**DOI:** 10.1186/s12917-026-05829-6

**Published:** 2026-08-18

**Authors:** Maciej Zdun, Aleksander F. Butkiewicz

**Affiliations:** 1 Department of Basic and Preclinical Sciences, Nicolaus Copernicus University in Torun, Lwowska 1, Toruń, 87‑100 Poland; 2 Department of Animal Anatomy, Poznan University of Life Sciences, Wojska Polskiego 71C, Poznan, 60-625 Poland

**Keywords:** Anatomy, Brain base arteries, Rete mirabile, RERM

## Abstract

**Background:**

Studies on the blood vessels of the brain in Cameliformes have largely focused on Old World camels, with limited data on New World species such as alpacas. The rostral epidural rete mirabile, a specialized arterial network at the base of the brain, plays a role in brain cooling, water conservation, and neurotransmitter transport in other ungulates. Alpacas are increasingly bred in Europe yet detailed anatomical descriptions of their cerebral arteries and the rostral epidural rete mirabile are lacking. This study aimed to analyze the blood supply to the encephalon in alpacas, with a focus on the rostral epidural rete mirabile, to provide insights relevant for anatomy, physiology, and veterinary care.

**Results:**

The internal carotid artery enters the rostral epidural rete mirabile from the caudal side, accompanied by branches from the maxillary and external ophthalmic arteries. The paired rete is well-developed, located on the basal sphenoid bone, with 51–64 interconnecting vessels forming an elongated oval structure. Blood from the rete supplies the cerebral arterial circle, including the rostral cerebral artery and caudal communicating arteries. The rostral cerebral arteries are connected rostrally by the rostral communicating artery in most specimens. The caudal cerebral and cerebellar arteries, along with the basilar artery, supply the posterior cerebrum, brainstem, and cerebellum. Variations were observed in the presence and branching patterns of certain arteries, including unilateral or bilateral absence of caudal branches. The rete is embedded in the cavernous sinus, which has a spongy structure due to interspersed arterial vessels.

**Conclusions:**

In alpacas, the brain is supplied by the internal carotid artery, the rostral and caudal branches to the rostral epidural rete mirabile, and the basilar artery. The vascular pattern remains constant during ontogenesis, with the internal carotid artery preserved in adults. The rostral epidural rete mirabile is well developed, especially in its rostral part, and embedded within the cavernous sinus. The cerebral arterial circle is formed by the rostral cerebral and caudal communicating arteries. The basilar artery plays a key role in maintaining adequate blood flow to the brain.

## Background

There have been numerous studies on the blood vessels of the encephalon and its tributaries in animals of the Cameliformes suborder, which belongs to the Artiodactyla order. However, researchers have usually described the dromedary [1–9] and Bactrian camel [5], which are camelids of the Old World. Researchers have also described some New World camelids such as the llama and guanaco [5]. O’Brien [10] used four specimens of the alpaca to describe the arteries in the head and neck, but focused mainly on the facial vessels. The researcher superficially listed the arteries supplying blood to the encephalon but did not describe all vessels supplying blood.

Detailed knowledge of the cerebral vascular anatomy is important not only from the perspective of normal anatomy. Cerebral vessels may serve as additional anatomical landmarks facilitating the precise identification of cerebral sulci and gyri. Moreover, a comprehensive understanding of the cerebral vascular system is essential for elucidating the pathophysiological mechanisms underlying various diseases, including disorders emerging with the advancement of modern veterinary medicine. Accurate characterization of cerebral blood supply pathways may provide a foundation for further studies on cardiovascular diseases and their consequences, contributing to a better understanding of their etiology, progression, and potential clinical implications. Blood supply pathways to the brain are of particular importance in clinical situations associated with impaired cerebral perfusion, such as vascular congestion or syncope resulting from insufficient blood flow. The brain is highly susceptible to hypoxia and lacks the ability to store glucose or glycogen; therefore, maintaining an adequate blood supply is essential for its proper function [11–15].

The rostral epidural rete mirabile is located in the base of the cranial cavity. It is composed of a tight network of anastomosed arteries occupying the whole cavity of the cavernous sinus. It is H-shaped and composed of two lobes on each side of the hypophysis. Each lobe is divided into two parts: the rostral and caudal part. The shape of the unilateral lobe is an elongated oval. It is a characteristic structure for Artiodactyla [3, 16, 17].

Since the 1970s scientists have been analysing the structure and significance of the rostral epidural rete mirabile in ruminants, which is another suborder of even-toed ungulates [18]. There have been similar studies conducted on camels [19]. So far researchers have described the role of the rostral epidural rete mirabile in selective cooling of the brain [18–21], the conservation of body water [22], retrograde transport of neurotransmitters [23, 24], and weakening the pulse wave and protecting the encephalon from sudden pressure surges [25]. As far as the Cameliformes are concerned, Jerbi et al. [3] described the rostral epidural rete mirabile of the camel with more details, specifying its dimensions and relations with the cavernous sinus. Due to the fact that the alpaca is increasingly often bred or kept as a companion animal in Europe, Australia, and North America, any information on this species may be useful for its breeding and treatment. Originally the alpaca inhabited the Andes in South America [26]. When transferred to regions with higher humidity and temperature, alpacas may experience heat stress [27]. As there has been no detailed description of the arteries of the encephalon base and the vessels supplying blood to this vascular region of the alpaca, the aim of this study was to analyse the blood vessels of the encephalon with a special focus on the rostral epidural rete mirabile. This information may be useful in physiological studies as well as studies on the welfare of these animals.

## Materials and methods

The material for the study consisted of 41 adult alpacas (15 males and 26 females) and four foetuses with a crown–rump length (CRL) of 182–254 mm, all belonging to the Cameliformes suborder. The cadavers were obtained from polish zoos and private breeders with the consent of the respective owners. Additionally, part of the material consisted of long-term collections maintained by the university. The animals were euthanized or died for reasons other than neurological diseases. Euthanasia was performed by practicing veterinarians and not by the authors of the study. No animal was euthanized for the purposes of this study. Ethical review and approval were waived for this study because the experimental work was conducted on cadavers. All procedures involving cadavers in accordance with the law of 15 January 2015 on the protection of animals used for scientific or educational purposes do not require the approval of the local ethics committee.

Twenty-three specimens were prepared by injecting red self-curing DURACRYL^®^ PLUS (SpofaDental, Jičín) into the bilateral common carotid arteries. Blue DURACRYL^®^ PLUS was also injected into the bilateral external jugular veins of 4 specimens. After curing for approximately 20 min, the specimens were enzymatically macerated with Persil powder (Henkel, Düsseldorf) at 42 °C for around one month, resulting in corrosion casts of the blood vessels on the skeletal framework. The remaining 20 specimens were prepared by injecting liquid red LBS 3060 latex into the bilateral common carotid arteries. In addition, liquid blue latex LBS 3060 was introduced into the bilateral external jugular veins of 4 specimens. After curing in 5% formalin solution for 14 days, the blood vessels were manually prepared using surgical instruments, thereby isolating the vascular structures from the surrounding soft tissues.

Two alpaca cadavers were used for the angio-CT examination. The specimens were frozen within one hour after death to ensure optimal tissue preservation, and scanning was performed immediately after thawing. Prior to imaging, barium sulfate (barium sulfuricum 1.0 g/mL, Medana, Sieradz, Poland) was injected into both common carotid arteries to enhance the visualization of blood vessels. The heads were securely positioned on the tomography table to prevent any movement during the procedure. Cone-beam computed tomography (CBCT) was used for imaging, with the following parameters: voltage 110 kVp, exposure 0.08 mAs per frame, total exposure 20.48 mAs, and reconstructed slice thickness 0.29 mm.

The photographs were taken with a Nikon D3200 digital camera and saved in JPG format. The resulting images were then processed and refined using the digital editing software Preview (Version 11.0, 1069.7.1; Copyright © 2002–2024, Apple Inc., Cupertino, CA, USA).The names of the anatomical structures were standardised according to the Nomina Anatomica Veterinaria [28].

## Results

The following observations apply to all specimens, regardless of age and sex.

### The internal carotid artery

**Fig. 1:**
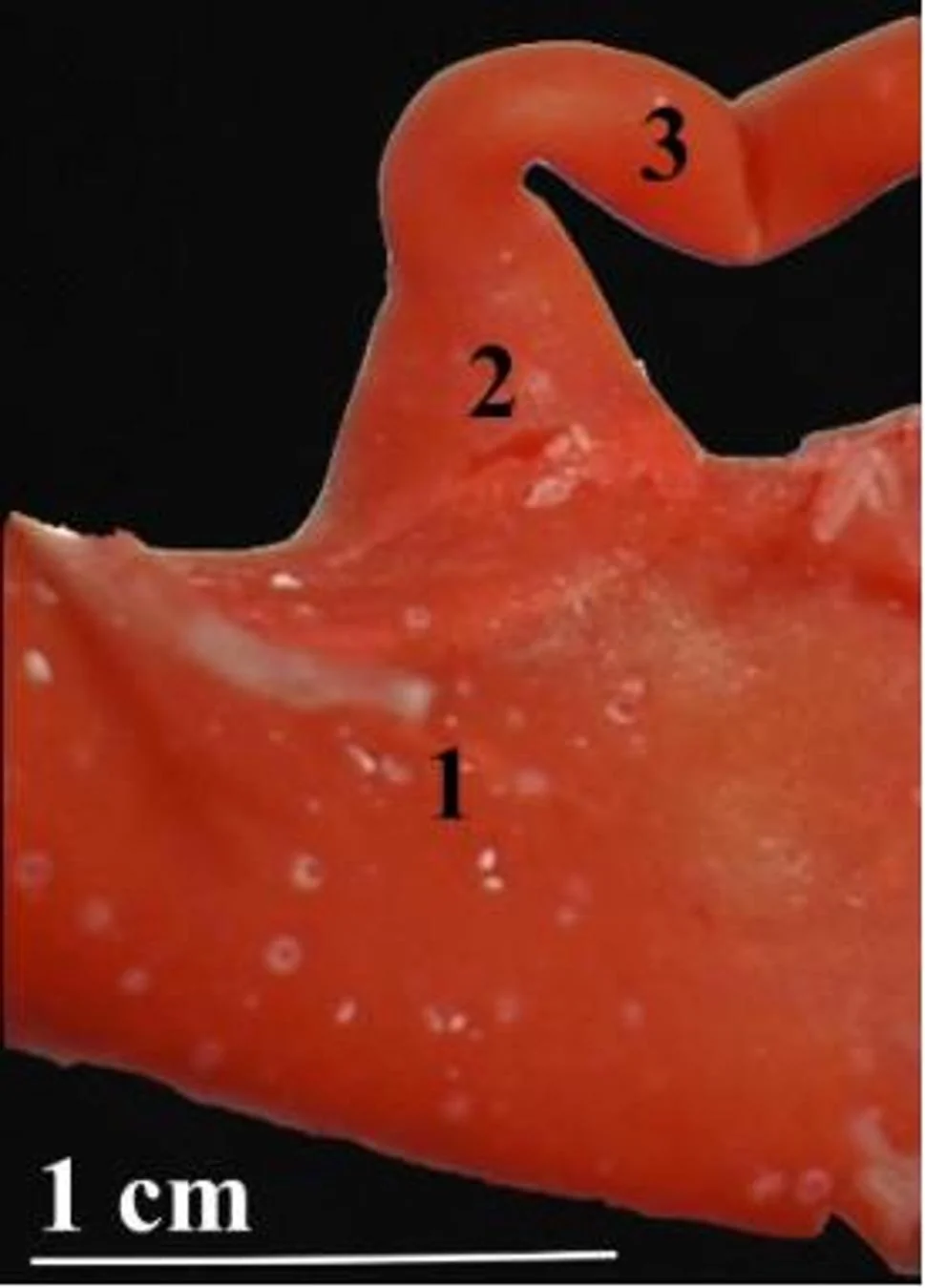
Carotid sinus. Lateral view of the corrosion cast. 1 – the common carotid artery; 2 – the carotid sinus; 3 – the internal carotid artery

Blood flows from various directions into the arteries on the encephalon base. The internal carotid artery (*arteria carotis interna*) branches at the site where the common carotid artery (*arteria carotis communis*) changes into the external carotid artery (*arteria carotis externa*). Having passed through the carotid canal, it enters the rostral epidural rete mirabile from the caudal side of the rete. Before joining the rete the lumen of the internal carotid artery is slightly smaller than at the point of branching from the common carotid artery. Carotid sinus (*sinus caroticus*) was observed at the beginning of the course of the internal carotid artery (Fig. 1). Before joining the rostral epidural rete mirabile the internal carotid artery bends slightly in an S-shaped manner. This vessel was found in all the specimens, regardless of age.

### Branches to the rostral epidural rete mirabile

**Fig. 2:**
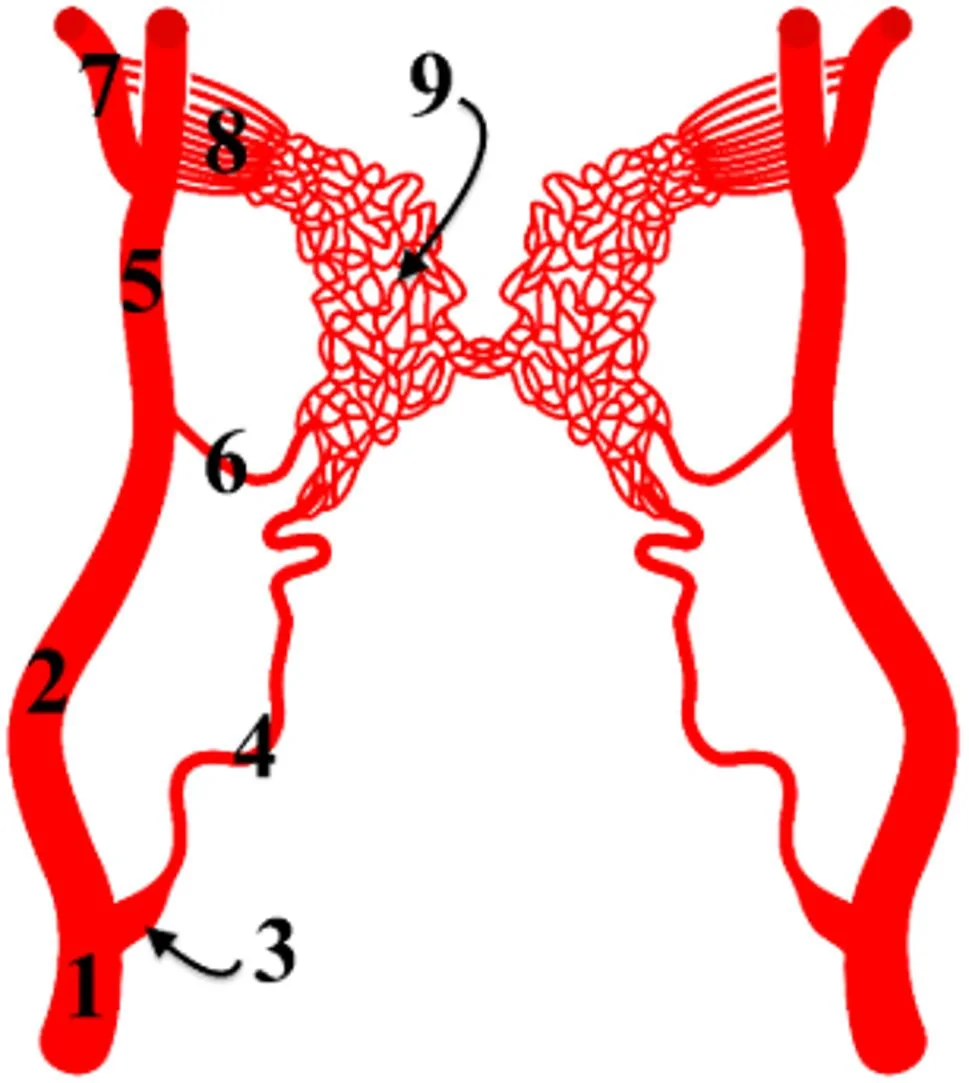
Diagram of drainage to the rostral epidural rete mirabile. 1 – the common carotid artery; 2 – the external carotid artery; 3 – the carotid sinus; 4 – the internal carotid artery; 5 – the maxillary artery; 6 – the caudal branch to the rostral epidural rete mirabile; 7 – the ophthalmic artery; 8 – the rostral branches to the rostral epidural rete mirabile; 9 – the rostral epidural rete mirabile

Blood is also supplied to the rete mirabile through the numerous rostral branches to the rostral epidural rete mirabile (*rami rostrales ad rete mirabile epidurale rostrale*), branching from both the maxillary artery (*arteria maxillaris*) and the external ophthalmic artery (*arteria ophthalmica externa*). There were much more vessels branching from the maxillary artery (8–17) than from the external ophthalmic artery (2–3). There was also a very thin caudal branch to the rostral epidural rete mirabile (*ramus caudalis ad rete mirabile epidurale rostrale*) (Fig. 2). However, two specimens lacked this vessel unilaterally and six specimens bilaterally.

### The rostral epidural rete mirabile

**Fig. 3:**
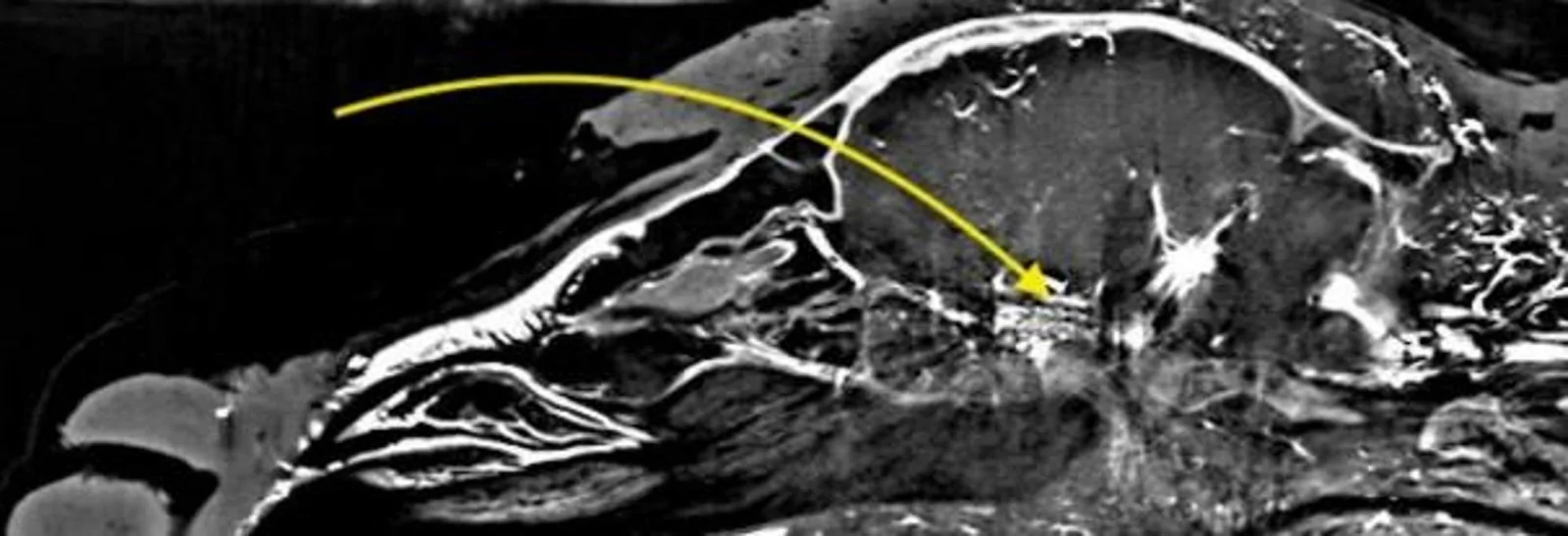
Maximum intensity projection of the agioCT scan of the head of the alpaca. Saggital plane. The rostral epidural rete mirabile in the cranial cavity

**Fig. 4:**
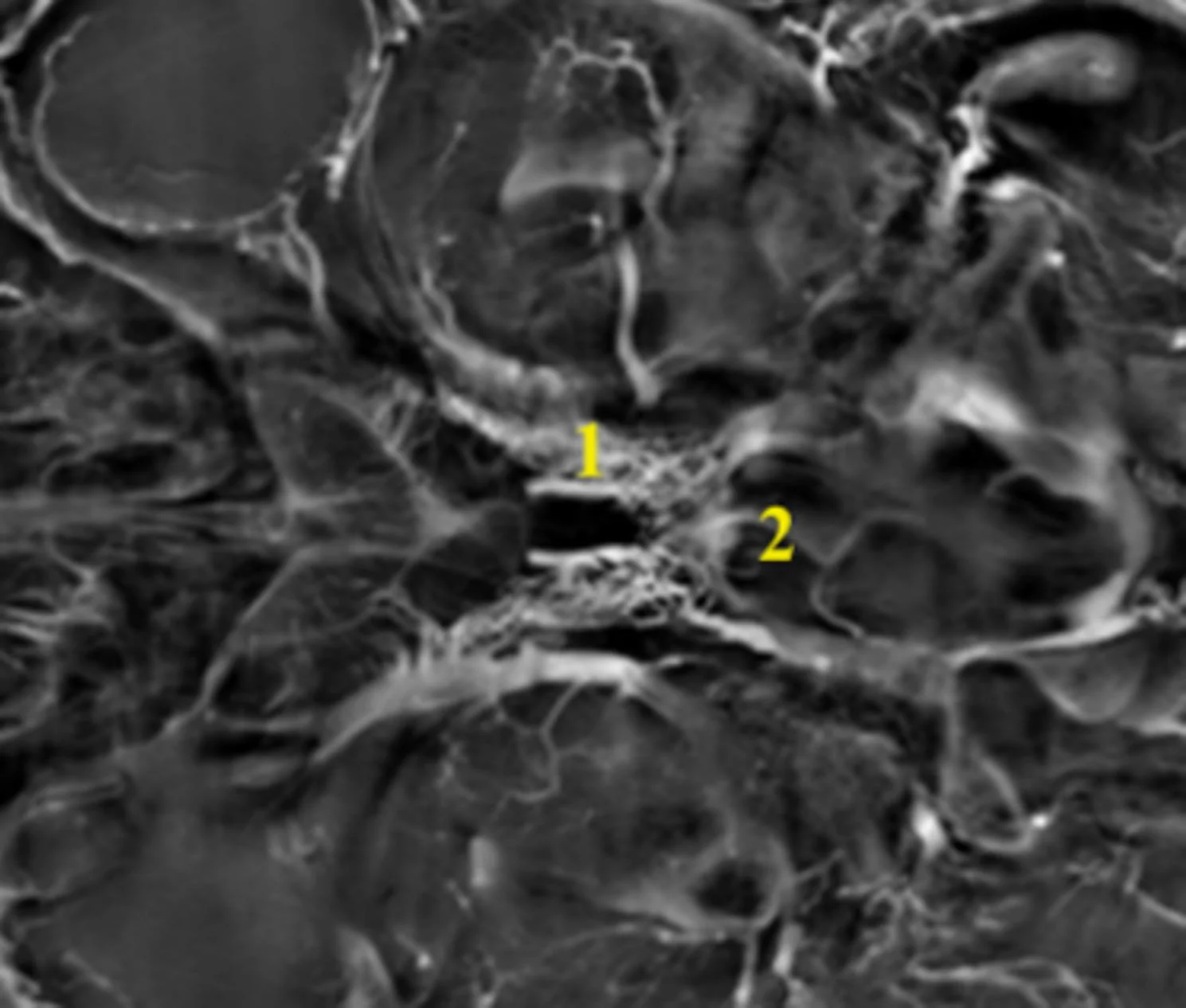
Maximum intensity projection of the agioCT scan of the head of the alpaca. Axial plane. 1 – the rostral epidural rete mirabile; 2 – the basilar artery

The rostral epidural rete mirabile is a well-developed paired structure, located on the body of the basal sphenoid bone, on the sides of the pituitary fossa (Figs. 3 and 4). It is composed of numerous multiply anastomosing arteries. Its shape resembles an elongated oval, which ends gently in the caudal section. The cranial section of the rete is asymmetrical. It is much more developed on the lateral side due to the rostral branches, which join the rete at this point. The cross section of the rete showed that it was flattened, with a plexus of tiny vessels of similar diameter. There were 51–64 vessels in the rete in adults, but on average, there were six more vessels in the right rete than in the left one. In fetuses the number of vessels in the cross-section was not counted. There were relatively few connections between the bilateral retia in the caudal section. Together the bilateral retia were H-shaped. Apart from that, there was a single vessel branching in the horizontal plane from the caudal end of the rete mirabile. It was connected to the same vessel on the opposite side. In the midline an odd artery branched caudally from this arcuate vessel and formed a Y-shaped structure from which tiny vessels branched.

### The arterial circle of the brain

**Fig. 5:**
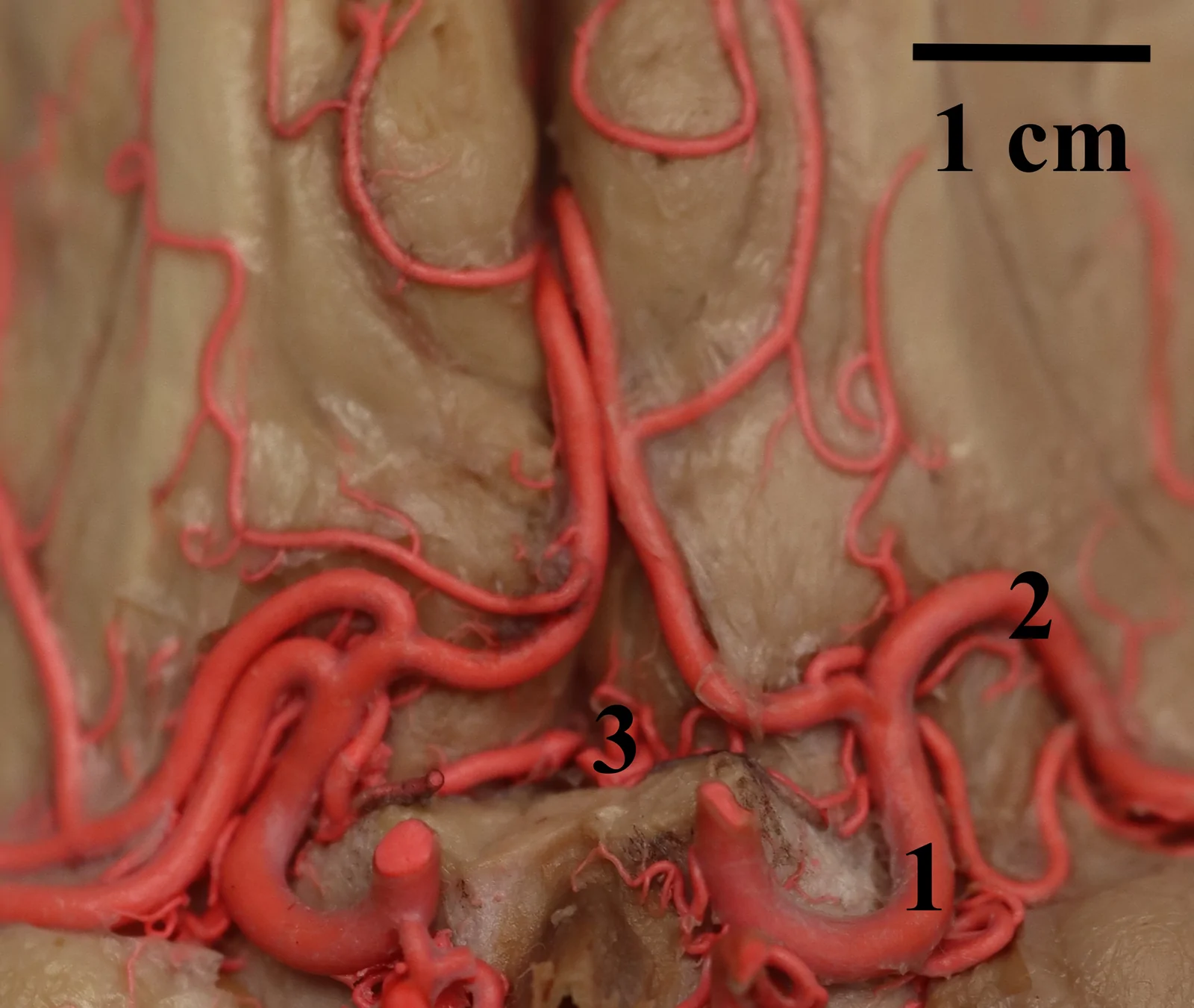
The rostral part of the arterial circle of the brain. Latex preparation. 1 – the rostral cerebral artery; 2 – the middle cerebral artery; 3 – the rostral communicating artery

The following vessels emerge from the rostral epidural rete mirabile: the rostral cerebral artery (*arteria cerebri rostralis*) and the caudal communicating artery (*arteria communicans caudalis*), which are the main components of the cerebral arterial circle (*circulus arteriosus cerebri*). Given their diameter, these vessels are the main source of blood for the encephalon, especially for the cerebrum, but they also supply large amounts of blood to the cerebellum. The rostral cerebral artery forms the rostro-lateral part of the cerebral arterial circle. It emerges from the rete mirabile at the site of the medial extremity of the lateral Sylvian fissure, lateral to the optic chiasm. There are two parts in the course of this vessel. The first segment runs horizontally along a flattened C-shaped arc from the site where this vessel is formed to the point where the longitudinal cerebral fissure begins. The bilateral rostral cerebral arteries are connected rostrally from the optic chiasm by the rostral communicating artery (*arteria communicans rostralis*) (Fig. 5).

The cerebral arterial circle is closed at the rostral side, by the rostral communicating artery. Seven of the specimens analysed in our study lacked the rostral communicating artery. The second segment begins at the point where the previous one ends, but the vessel changes its course and runs dorsally along the longitudinal cerebral fissure. Next, it runs caudally along the median plane of the frontal and parietal lobes of the cerebral hemisphere and reaches the dorsal surface of these lobes. The middle cerebral artery (*arteria cerebri media*) branches from the rostral cerebral artery at about half the length of its first segment. Our analysis showed 3 cases where this vessel branched with two legs bilaterally and 3 cases where it branched with two legs unilaterally. This artery surrounds the piriform lobe from the rostral side and then bends dorsally and supplies blood to the lateral surface of the frontal, parietal, and temporal lobes. Another vessel branching from the rostral cerebral artery is the rostral choroidal artery, which runs laterally and then dorsally between the temporal lobe and cerebral crus.

**Fig. 6:**
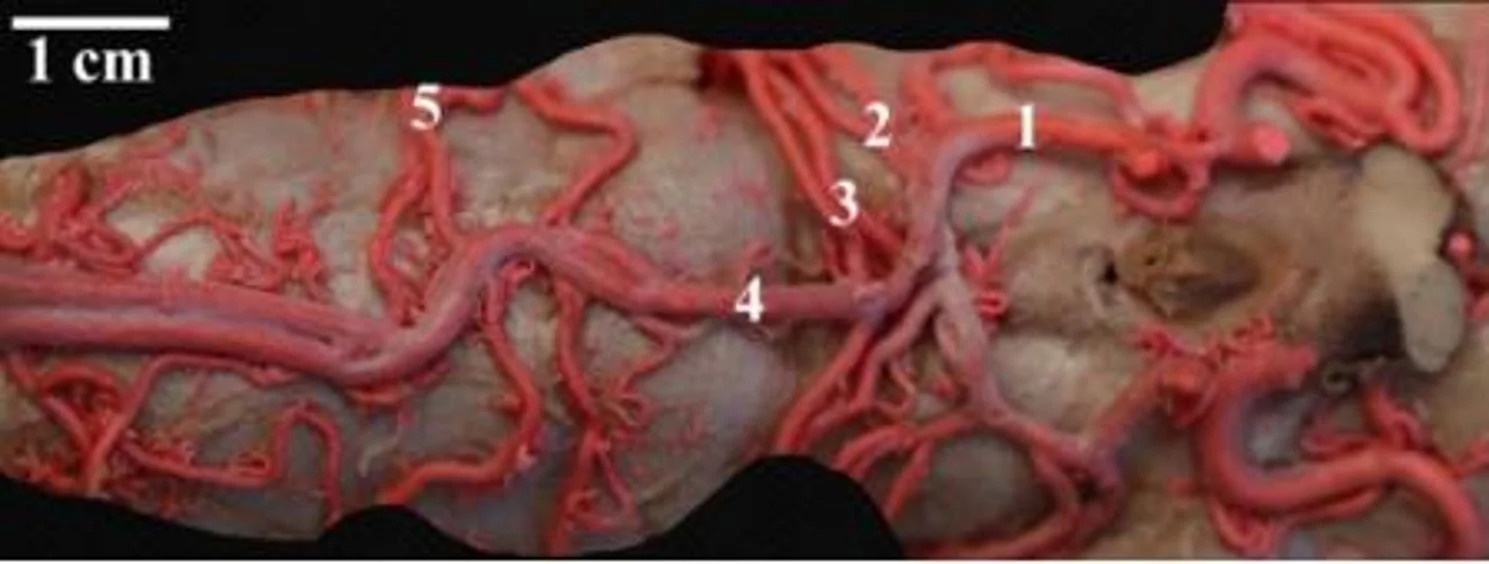
The caudal part of the arterial circle of the brain. Latex preparation. 1 – the caudal communicating artery; 2 – the caudal cerebral artery; 3 – the rostral cerebellar artery; 4 – the basilar artery; 5 – the caudal cerebellar artery

**Fig. 7:**
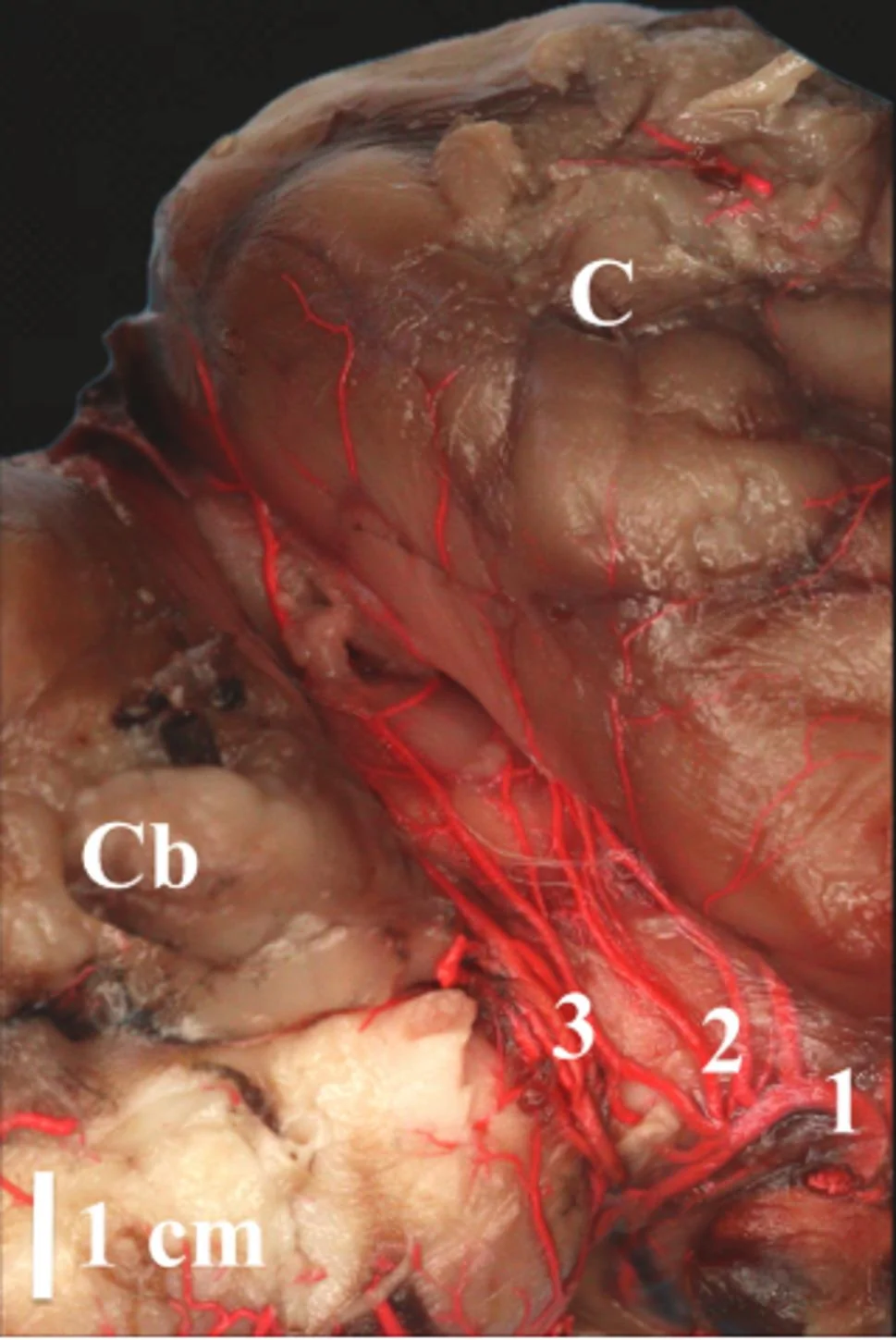
The caudal part of the arterial circle of the brain. Latex preparation. C - cerebrum Cb - cerebellum 1 – the caudal communicating artery; 2 – the caudal cerebral artery; 3 – the rostral cerebellar artery

In the caudal section, the cerebral arterial circle consisted of paired caudal communicating arteries (Fig. 6). They run along an arc which is less curved (more gentle) than the one along which the rostral cerebral arteries run. Bilateral vessels meet at the point where the odd basilar artery (*arteria basilaris*) joins them. The caudal communicating artery curves around the mammillary body and converges medially in the interpeduncular fossa. The caudal cerebral artery (*arteria cerebri caudalis*) (Fig. 7) branches from the caudal communicating artery. Among the vessels branching from the caudal communicating artery this one has the largest diameter. There was one specimen where the caudal cerebral artery was a double vessel unilaterally and four specimens where it was a double vessel bilaterally. The caudal cerebral artery runs close to the rostral choroid artery. It supplies blood to the cortical region of the occipital, parietal, and temporal lobes of the cerebrum. It runs medially and supplies blood to the medial and dorsal surfaces of the occipital and parietal lobes. Apart from that, numerous tiny vessels branch from the caudal communicating artery to supply blood to the cerebral peduncles of the midbrain.

The rostral cerebellar arteries (*arteriae cerebelli rostrales*) branch from the final segment of the caudal communicating artery and from the initial segment of the basilar artery, i.e. from the site where these vessels meet. There are 3–5 of them. They run dorsocaudally and supply blood to the anterior part of the cerebellum. The basilar artery *(arteria basilaris)* is a single, winding vessel coursing along the ventral surfaces of the medulla oblongata and pons. At the site where it meets the caudal communicating arteries its diameter is slightly smaller than in the more caudal segment, at the level of the impression of the medulla oblongata of the occipital bone, where it becomes a paired vessel. The caudal cerebellar artery *(arteria cerebelli caudalis*) also branches from the basilar artery. It is a single vessel on either side of the body. It branches perpendicular to the basilar artery at the caudal end of the pons. The caudal cerebellar artery communicates with the lateral branch of the vertebral artery and further anastomoses with the medullary branches of the basilar artery to supply blood to the medulla oblongata. It also anastomoses with the rostral cerebellar artery. The caudal cerebellar artery runs in the superior caudal direction to supply blood to the caudal part of the cerebellum. As was mentioned above, the basilar artery becomes a binary vessel in the caudal part. Each of its branches is formed by connection with the ventral spinal artery and the medial branch of the vertebral artery. The vertebral artery passes through the transverse foramen of the atlas and connects to the occipital artery. Then it passes through the lateral vertebral foramen. It splits into a weaker lateral branch and a stronger medial branch in the spinal canal. The medial branch connects to the ventral spinal artery near the greater foramen and thus forms the basilar artery in the rostral direction. The collected vascular variations are presented in Table 1.

**Table 1:** Anatomical variations of cerebral vessels (*n* = 45)

Vascular structure	Anatomical variation	Number of specimens	Frequency (%)
*Ramus caudalis ad rete mirabile epidurale rostrale*	Unilateral absence	2	4.4
*Ramus caudalis ad rete mirabile epidurale rostrale*	Bilateral absence	6	13.3
*Arteria communicans rostralis*	Absence of the vessel closing the cerebral arterial circle rostrally	7	15.6
*Arteria cerebri media*	Bilateral duplication (two branches)	3	6.7
*Arteria cerebri media*	Unilateral duplication (two branches)	3	6.7
*Arteria cerebri caudalis*	Unilateral duplication	1	2.2
*Arteria cerebri caudalis*	Bilateral duplication	4	8.9

### The cavernosus sinus

**Fig. 8:**
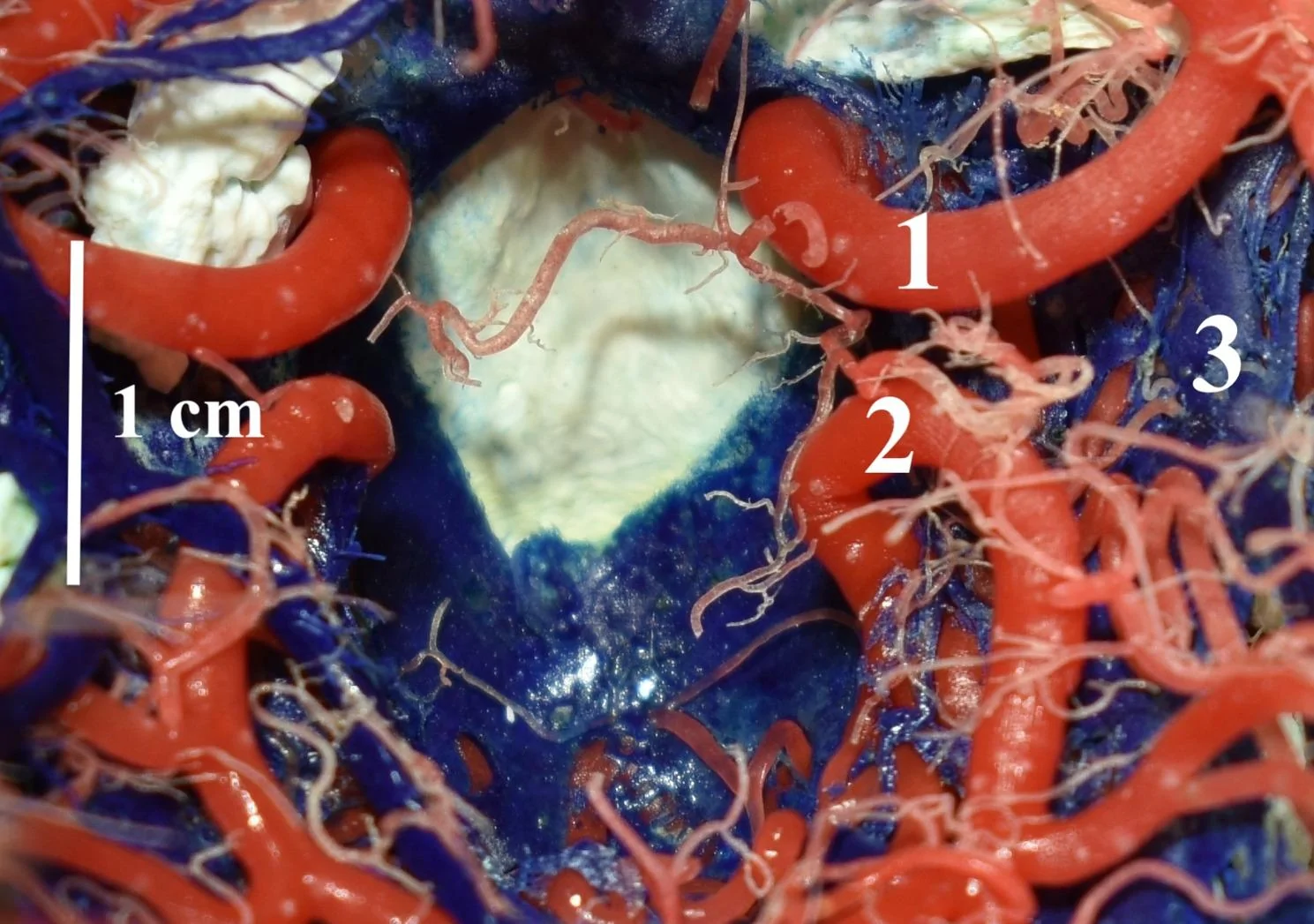
The cavernosus sinus. Dorsal view of the corrosion cast. 1 – the rostral cerebral artery; 2 – the caudal communicating artery; 3 – the cavernosus sinus

The arteries are not accompanied by homonymous veins. However, the arteries of the paired rostral epidural rete mirabile are accompanied by the venous vessels of the cavernous sinuses (*sinus cavernosus*). The rostral epidural rete mirabile is embedded in the cavernous sinus. The sinus has a spongy structure due to numerous small spaces filled with vessels of the arterial rostral epidural rete mirabile (Fig. 8).

## Discussion

In camelids blood is supplied to the rostral epidural rete mirabile mainly by the rostral rami to the rostral epidural rete mirabile branching from the maxillary artery and the external ophthalmic artery [1, 3, 5, 29]. Among ruminants, which, apart from Cameliformes, is another suborder of even-toed ungulates, this branching pattern was also observed in the Cervidae [30]. In bovids, which are also ruminants, the rostral rami to the rete branch off only from the maxillary artery [31]. Apart from that, there is a weak caudal ramus to the rostral epidural rete mirabile, which was identified as the middle meningeal artery by Al Aiyan et al. [32]. This vessel was not listed by Ocal et al. [29], Kiełtyka-Kurc et al. [5], or Jerbi et al. [3]. The caudal ramus to the rostral epidural rete mirabile has been described in all ruminants. It was relatively thick in bovids, but it was a small-calibre vessel in the Cervidae [30, 31]. Blood is also supplied to the rostral epidural rete mirabile through the internal carotid artery, which was described in the Bactrian camel and dromedary, llama, alpaca, and guanaco [1–3, 5, 10, 29]. Camelids are the only suborder of even-toed ungulates in which this artery is fully preserved. As far as the other suborders of even-toed ungulates are concerned, the internal carotid artery can be found only in foetuses and young animals. The vessel becomes obliterated as the body develops [33–36]. The only exception is the Siberian musk deer of the Moschidae family, in which the internal carotid artery is not obliterated [37]. The intracranial segment of the internal carotid artery has a tortuous course. Ruedi [38] analysed the course of this vessel in the horse and concluded that this segment was responsible for weakening the pulse wave and protecting the encephalon from sudden pressure surges. A similar role was assigned to the rostral epidural rete mirabile in ruminants [25]. If the rostral epidural rete mirabile serves this function, it is intriguing why the internal carotid artery of the alpaca also has a tortuous course in its rostral section despite the presence of the rete mirabile.

The shape of the rostral epidural rete mirabile presented in our study is consistent with the observations made by the authors who described the rete in other camelids. The most detailed description of the rete in camelids was provided by Jerbi et al. [3], who studied the dromedary. The lateral side of the rostral part of the rete is particularly well-developed. The rete has rostral and caudal compartments in relation to the hypophysis. The rostral compartment represents the main part of the rete mirabile [3]. Although in comparison with the retia of ruminants, the cross-section did not reveal a large number of arteries in the rete [31], its anterior part was much better developed, which resulted in a larger contact surface between the arterial and venous systems. It is particularly important for the rete mirabile vessels to contact the cavernous sinus because this contact enables the rete to perform several important functions. Research has shown that the encephalon physiologically exhibits relatively large temperature fluctuations within 2–4 °C, resulting from the natural life activity. Temperature fluctuations may be even greater in the camel – even up to 7 °C [39]. Apart from the observed physiological hyperthermia, excessively high temperature in the encephalon may cause abnormalities and negatively influence the nervous functions and activity [40]. Hayward and Backer [18] described the mechanism of selective cooling of the brain, which consists in cooling warmer arterial blood flowing to the encephalon. Cooled blood flows from the nasal mucosa to the cavernous sinus. As a result, the temperature of the blood flowing to the encephalon is lowered more effectively [18, 20, 21, 34]. The course of the vessels enables the animal to maintain the encephalon temperature at 40.5 °C during muscular effort. Experiments conducted on the Thomson’s gazelle showed that the temperature of its encephalon was 3.25 °C lower than the temperature of its blood in the common carotid artery [41]. A similar study conducted on domestic cattle showed a temperature difference of 1.3 °C [20], whereas a study on camels showed a difference of 3.98 °C [42]. This phenomenon is a particularly important thermal adaptation of large mammals inhabiting arid climatic zones [43]. Research confirmed the existence of selective cooling of the brain in camels. Under normal conditions, after the cool venous blood has passed through the nasal cavity, it travels via a general circulation. However, when the body temperature increases, the nasal and angular veins become wider, whereas the facial vein is constricted, which increases selective cooling of the brain [44]. The sphincters in the walls of the vessels regulate the blood flow by responding to stimulation of the sympathetic system. Alpha receptors can be found in the nasal and angular veins, whereas beta receptors can be found in the facial vein [19, 44].

Researchers also observed selective cooling of the brain at night, when temperatures were not extremely high [45]. Studies have confirmed the fact that selective cooling of the brain not only affects the thermoregulation of the encephalon but also significantly influences the body water-saving mechanism, because it enables adaptation to an extremely hot and dry environment [22]. The water-saving mechanism was researched on Arabian oryxes living in Saudi Arabia. The mechanism of selective cooling of the brain in representatives of this species was compared with the same phenomenon observed in the gemsbok living in areas with similar temperatures but less dry. Experiments showed that the mechanism of selective cooling of the brain in Arabian oryxes was more efficient than in gemsboks [22]. A study on Dorper sheep showed that animals weighing about 50 kg were capable of saving 2.6 l of water daily [46]. Selective cooling of the brain is a mechanism protecting animals from the loss of water. The hypothalamic stimulation of animals to sweat and pant is reduced by maintaining the brain temperature at a lower level than the rest of the body during periods of dehydration, which results in less evaporative cooling [47]. Research on sheep showed that despite exposure to high temperatures, selective cooling of the brain did not occur if the animals had access to water. However, the phenomenon occurred more frequently if the animals’ access to water was limited [48].

Apart from that, the rete mirabile and cavernous sinus are involved in the retrograde transport of neurotransmitters. Studies on pigs and sheep showed that dopamine [49, 50], beta-endorphin, GnRH, progesterone or testosterone [23], and oxytocin [51] diffuse from the venous blood of the cavernous sinus to the arterial blood of the rostral epidural rete mirabile. There are differences resulting from the oestrus cycle. The countercurrent transfer of beta-endorphin takes place during the early luteal phase in the breeding season, but not during seasonal anoestrus. Studies showed that the efficiency of the permeability of oxytocin [51] and LHRH in pigs [24, 52] depends on the day of the oestrous cycle. Moreover, the cavernous sinus system in the rete mirabile transports the regulators of neurogenic carbon monoxide (CO). The energy from visible light stimulates the production of carbon monoxide by the retina, which penetrates through the cavernous sinus into the vessels of the rostral epidural rete mirabile, and causes changes in the activity of the brain [53]. Being a physiological regulator, carbon monoxide has a decisive influence on the brain function as the main biological clock. It alters the expression of the *per* and *cry* genes, which modulate the circadian rhythm and seasonal cycles [54]. The comparative analysis of the structure of the retia in the families under analysis allows us assume that the rete mirabile plays a similar role in the alpaca. In the cross-section the rostral epidural rete mirabile was a plexus of tiny vessels of similar diameters. Simoens et al. [34] referred to such a rete as bipolar.

It is characteristic of camelids that their rostral cerebral artery and caudal communicating artery emerge independently right from the vessels of the rostral epidural rete mirabile [1, 3, 5, 7, 8]. In the other suborders of even-toed ungulates these vessels have a common short trunk and then they divide into the above-mentioned vessels [35, 36, 55–58]. The course and area to which blood is supplied by the arteries branching from the rostral cerebral artery and caudal communicating artery is consistent with the description provided by Al Aiyan et al. [1, 7–9], who conducted a study on the camel. The basilar artery deserves attention. In the alpaca the diameter of the basilar artery at the site where it meets the caudal communicating arteries is slightly smaller than in the more caudal segment. The situation is similar in the camel [2]. On the other hand, in ruminants the caudal segment near the connection with the vertebral arteries is thinner than its rostral segment, which anastomoses with the cerebral arterial circle. This property of the basilar artery in domestic cattle and domestic sheep inspired Baldwin and Bell [59–61] to conduct an experiment on the flow of blood to the encephalon of these animals. The research results showed that the basilar artery was not involved in supplying blood from the vertebral arteries to the encephalon. This property of the basilar artery in ruminants was confirmed by the results of morphological investigations on representatives of the Antilopinae subfamily [55], the Caprinae subfamily [62, 63], species of the nilgai, nyala and common eland genera [56], northern giraffe [64], red deer [57] and roe deer [58]. The study conducted by Al Aiyan et al. [2] proved that a very large amount (34.6%) of blood was supplied through the basilar artery to the camel’s encephalon. Therefore, it is necessary to note that this vessel has a completely different role in the distribution of blood to the encephalon of camelids and ruminants.

## Conclusions

1. Blood is supplied to the alpaca’s encephalon through the internal carotid artery, the rostral rami and caudal ramus to the rostral epidural rete mirabile and the basilar artery.
2. The vessels supplying blood to the encephalon remain unchanged during ontogenesis. The internal carotid artery does not become obliterated but it is also preserved in adult animals.
3. The rostral epidural rete mirabile is a very well-developed structure, especially its rostral segment. The vessels of the rete are embedded in the cavernous sinus.
4. The cerebral arterial circle is composed of the rostral cerebral arteries and caudal communicating arteries.
5. The basilar artery is an important source of blood for the encephalon.

## Data Availability

The data that support the findings of this study are not openly available and are available from the corresponding author upon reasonable request.
